# Effect of Pelagic *Sargassum* on In Vitro Dry Matter and Organic Matter Degradation, Gas Production, and Protozoa Population

**DOI:** 10.3390/ani13111858

**Published:** 2023-06-02

**Authors:** Luis Alberto Canul-Ku, José Roberto Sanginés-García, Edgar Aguilar Urquizo, Jorge Rodolfo Canul-Solís, Ingrid Abril Valdivieso-Pérez, Einar Vargas-Bello-Pérez, Isabel Molina-Botero, Jacobo Arango, Ángel Trinidad Piñeiro-Vázquez

**Affiliations:** 1 Tecnológico Nacional de México, Instituto Tecnológico de Conkal, Conkal 97345, Mexico; 2 Tecnológico Nacional de México, Instituto Tecnológico de Conkal, Tizimín 97000, Mexico; 3 Department of Animal Sciences, School of Agriculture, Policy and Development, University of Reading, P.O. Box 237, Earley Gate, Reading RG6 6EU, UK; 4 Facultad de Zootecnia y Ecología, Universidad Autónoma de Chihuahua, Periférico R. Aldama Km 1, Chihuahua 31031, Mexico; 5 The Alliance of Bioversity International and the International Center for Tropical Agriculture (CIAT), Km 17 Recta Cali-Palmira, Cali A.A. 6713, Colombiaj.arango@cgiar.org (J.A.)

**Keywords:** brown algae, secondary metabolites, fermentation parameters, alternative ingredient, protozoa

## Abstract

**Simple Summary:**

Despite studies conducted with other seaweed species on in vitro gas production, until now, no studies have been reported on the use of pelagic *Sargassum* as potential ruminant feed. Thus, the objective of the present study was to determine the effect of *Sargassum* inclusion, using tropical grass as substrate, on in vitro gas production kinetics. Additionally, heavy metals and macro- and microminerals were determined in *Sargassum*. For that, in vitro incubations were performed with different levels of *Sargassum* inclusion on a basal substrate (Stargrass hay). In vitro results showed that up to 30% pelagic *Sargassum* could be included in hay-based substrates from tropical grasses.

**Abstract:**

This study determined the effect of pelagic *Sargassum* on in vitro dry matter and organic matter degradation, total gas production (TGP), and protozoa population. The treatments were different levels of *Sargassum* inclusion on a basal substrate (Stargrass hay; *Cynodon nlemfuensis*) as follows: T0 (control treatment based on Stargrass hay), T10 (90% Stargrass hay + 10% *Sargassum*), T20 (80% Stargrass hay + 20% *Sargassum*), and T30 (70% Stargrass hay + 30% *Sargassum*). Ruminal fermentation kinetics and protozoa population were determined during 72 h of in vitro incubations. Compared to control, dry matter degradability at 48 and 72 h and organic matter degradability at 24 and 48 h were higher in *Sargassum* treatments. TGP was lower with T20 at 48 h. The total population of protozoa and the concentration of *Entodinium* spp. were lower at T20 at 48 h and T30 at 72 h. Cl, S, Ca, K, and Zn (103, 5.97, 88.73, 285.70 g/kg, and 15,900 mg/kg) were high in *Sargassum*, reaching twice or even nine times higher than the contents in Stargrass (11.37, 1.60, 43.53, 87.73 g/kg, and 866.67 mg/kg). Overall, up to 30% pelagic *Sargassum* could be included in hay-based substrates from tropical grasses without negative effects on in vitro dry matter and organic matter degradability.

## 1. Introduction

The growing animal protein demand driven by the increase in the world population, and the adverse effects of change climate, put pressure on the agricultural production systems to increase its productive efficiency [1]. In relation to the negative impacts on the environment, greenhouse gas emissions (GHG) are the most related to ruminant production systems [2]. Within the GHG generated from livestock, the most important are methane (CH_4_), carbon dioxide (CO_2_), ammonium (NH_4_+), and nitrous oxide (N_2_O) [3,4,5]. GHG produced in the rumen can be mitigated through feeding strategies which, on the one hand, improve the digestibility of organic matter (OM); and on the other, modify the rumen microbiome [2,6,7] using secondary metabolites, by-products, and essentials oils.

In the Mexican Caribbean, one possible alternative is the use of seaweed as a source of bioactive substances that could help to reduce CH_4_ production through modulation of the ruminal microbiome [1,8,9]. *Sargassum* is found in relatively large amounts in the Mexican coasts of the Yucatan Peninsula, which represent a serious problem for the tourism sector, and this has led to the development of projects aiming to make use of this pollutant [10,11,12]. In this regard, pelagic *Sargassum* that reaches the coasts of the Mexican Caribbean is mainly composed by two species of algae brown: *Sargassum natans* and *Sargassum fluitans* [13]. These marine species could be alternative feeds that could improve OM fermentation in ruminants, as shown in in vitro studies with other species of marine algae [14,15,16]. The nutritional value of seaweed is related to its content of minerals, polysaccharides, and phenolics compounds such as phlorotannin that can modify ruminal microbiome toward reducing CH_4_ production [17,18,19,20,21]. Some in vitro studies conducted with brown and red seaweed have shown improvements on fermentation characteristics and reductions in CH_4_ [20,22,23,24]. However, until now, studies have not been conducted on the use of pelagic *Sargassum* as potential ruminant feed. Based on the above, the objective of the present study was to determine the effect of *Sargassum* inclusion, using a tropical grass as substrate, on in vitro gas production kinetics. Additionally, heavy metals and macro- and microminerals were determined in *Sargassum*.

## 2. Materials and Methods

### 2.1. Location

The study was carried out in the digestive physiology laboratory of the Technological Institute of Conkal, Yucatan, Mexico. Located at coordinates 21°04′45.9″ N 89°29′57.7″ W, at 7 m above sea level, with an Aw0 climate according to the Köppen climate classification, as modified by García (1988). The highest proportion of rainfall occurs during the months of June to October, with 900 mm of precipitation, an average annual temperature of 29 °C, and annual average relative humidity between 66 and 89%.

### 2.2. Management of Donor Animals

The animals were handled according to the animal handling and welfare standards of the Technological Institute of Conkal (project 15135). Five male lambs of the Pelibuey breed (four years of age) with a body weight of 40 ± 3 kg were used as donors of rumen contents. Lambs were fed solely on Stargrass (*Cynodon nlemfuensis*) from grazing paddocks. For 15 consecutive days, grazing had a daily duration of 8 h (from 8:00 am to 4:00 pm). After grazing, animals were housed in individual roofed pens (3 × 3 m) with free access to water. At the beginning of the adaptation period, the sheep were dewormed with Closantel 5%^®^ (Wyeth LLC, Madison, NJ, USA) at a dose of 10 mg/kg of body weight.

### 2.3. Experimental Design and Treatments

A completely randomized design with four treatments and four replications was used. In vitro gas production (IVGP) was recorded at 0, 3, 6, 9, 12, 24, 36, 48, and 72 h of incubation; while pH and ruminal protozoa population, dry matter degradation, and organic matter degradation, were analyzed at 24, 48, and 72 h of incubation. The treatments consisted of different levels of *Sargassum* inclusion on a basal substrate (Stargrass hay; *Cynodon nlemfuensis*) as follows: T0 (control treatment based on Stargrass hay); T10 (90% Stargrass hay + 10% *Sargassum*); T20 (80% Stargrass hay + 20% *Sargassum*); and T30 (70% Stargrass hay + 30% *Sargassum*).

### 2.4. Sample Preparation

The *Sargassum* was collected in September 2021 on the beach of San Miguelito, Municipality of Cancun, State of Quintana Roo, Mexico. Subsequently, the *Sargassum* was cut into small pieces and dried naturally in the shade and then ground in a Wiley mill (Thomas Wiley Laboratory Mill, Swedesboro, NJ, USA) with a sieve size of 2 mm to convert it into flour. The Stargrass was harvested on day 28 of growth and immediately dried in a forced air oven at 60 °C for 24 h and ground in a Wiley mill (Thomas Wiley Laboratory Mill, Swedesboro, NJ, USA) with a sieve size of 2 mm.

### 2.5. In Vitro Trial

Rumen contents (solid to semisolid phases) were obtained through an oesophageal probe as described by Ramos-Morales et al. [25] at 08:30 h before grazing. This was performed to reduce the variation of inoculum composition and activity and to minimize the influence of the diet fed to the donor animals [26]. Contents were kept in thermos at a constant temperature of 39 °C to be transferred to the digestive physiology laboratory where they were filtered through four layers of gauze to obtain only the liquid fraction, which was saturated with CO_2_ when mixed with reduced mineral solutions according to Menke and Steingass [26].

The tropical grass was used as the basal substrate in the four treatments with different percentages of inclusion of *Sargassum* that resulted from the mixtures of Stargrass hay and *Sargassum*. One gram of the mixture resulting from each treatment was placed in 48 amber glass bottles (*n* = 4 times), with a capacity of 100 mL [26]. In addition, 24 flasks were used as blanks (only with ruminal fluid) to correct for gas production. Once the vials with the samples were prepared, they were filled with the inoculum and sealed with their respective aluminum rings and rubber stoppers to be incubated in a water bath at 39 °C, and the pressure and gas volume readings were recorded at 3, 6, 9, 12, 24, 36, 48, and 72 h, in addition to the variables described below.

### 2.6. In Vitro Gas Production

The volume of gas generated (mL/g of incubated DM and OM) was measured according to the procedure proposed by Theodorou et al. [27], namely:

V = (P − 21.016)/16.132,

where:

V = volume of gas (mL);
P = measured pressure (psi).

To measure pressure changes, a pressure transducer was used. The kinetics of gas production was evaluated using the Gompertz model [28]:

Y = A exp {− exp [1 + be/A (LAG − t)]},

where:

Y = Cumulative total gas production (mL);
A = Theoretical maximum gas production (mL);
b = Maximum gas production rate (mL/h), which occurs at the inflection point of the curve;
LAG = Lag time (h), defined as the time axis intercept of the tangent line at the inflection point;
t = time.

Parameters a, b, and LAG were estimated by means of a non-linear regression analysis, for which the Origin 8 program was used. These parameters were used to evaluate the kinetics of gas production in vitro according to the methodology described by Machado et al. [26].

### 2.7. Fermentation Parameters

The inoculum was sampled to analyze pH and protozoa count at 24, 48, and 72 h of incubation. The pH was measured with a pH meter (ECOTESTR, Thermo Scientific Eutech Instruments, Mundelein, IL, USA). For protozoa count, 2 mL of the inoculum was taken after 24, 48, and 72 h of incubation and mixed with 2 mL of methyl green formalin saline solution, composed by 100 mL of 35% formaldehyde solution, 0.6 g of methyl green, 8.0 g of NaCl, and 900 mL of distilled water [29]. The protozoa were counted under the microscope using a counting chamber (Neubauer, Fuchs-Rosenthal). Each sample was counted six times, and when the mean of the repetitions differed by more than 10%, the counts were repeated.

### 2.8. Degradability

In vitro degradability of DM and OM was estimated using the digestion nylon bags method (Dacron^®^ fabric). After incubation, the nylon bag with the substrate was washed three times and then dried in an oven at 60 °C for 48 h. Subsequently, the bags were weighed to obtain the dry weight of the remaining sample and to obtain the DM degradability. The following formula was applied according to Choi et al. [20]:

$$Dry\;matter\;degradability\;\left(g\;kg^{-1}DM\right)=\frac{IDM\;weight-FDM\;weight}{IDM\;weight}\times 1000,$$

where:

*IDM* = initial dry matter;
*FDM* = final dry matter.

To determine OM degradability, the remaining samples of the nylon bags were incinerated in a muffle at 560 °C for 8 h to obtain the ash content and determine the OM of each incubated sample. Subsequently, the same formula was applied to calculate the OM degradability developed by Choi et al. [20].

$$Organic\;matter\;degratability\;\left(g\;kg^{-1}DM\right)=\frac{IOM\;weight-FOM\;weight}{IOM\;weight}\times 1000,$$

where:

*IOM* = initial organic matter;
*FOM* = final organic matter after incubation.

### 2.9. Chemical Analysis

Contents of dry matter (DM; method 934.01), crude protein (CP; method 954.01), crude fiber (CF; method 962.09), ethereal extract (EE; method 920.39), ash (AC; method 942.05) of *Sargassum*, and Stargrass hay were determined as described by the AOAC [30]. Neutral detergent fiber (NDF) and acid detergent fiber (ADF) were determined according to the procedures of Van Soest et al. [31]. Non-fibrous carbohydrates (NFC) were calculated as 100 − (CP + NDF + EE + Ash).

The determination and quantification of macronutrients (Cl, P, K, S, Ca, Mg, and Na), micronutrients (Mn, Fe, Zn, Cu, Al, and Si), and heavy metals (As) from *Sargassum* and Stargrass was performed by μ-X-Ray Fluorescence (μ-XRF) analysis, with the methodologies described by Morales-Morales et al. [32], using an M4Tornado 100 equipment (Bruker, Germany). The chemical and mineral composition of *Sargassum* and Stargrass are described in Table 1, Table 2 and Table 3.

### 2.10. Statistical Analysis

Data were analyzed by one-way analysis of variance (ANOVA), with the PROC GLM procedure for a completely randomized design in the SAS statistical software (SAS, 1999). In vitro gas production kinetics were analyzed using the Gompertz model [28]. Tukey’s test was used for comparisons of means between treatments. The results were considered statistically significant at a value of *p* < 0.05.

## 3. Results

### 3.1. Chemical Composition of Sargassum and Stargrass

Chemical composition revealed differences between the Stargrass hay and *Sargassum* mostly in CP contents (10.48 vs. 6.73%) and fiber fractions such as NDF (76.70 vs. 23.12%), and ADF (41.61 vs. 17.18%) (Table 1).

When mixing Stargrass hay and *Sargassum* (Table 2), it was observed that the concentration of ash increased as the level of *Sargassum* increased from 7.46 to 17.42%, and vice versa in the concentration of CP, OM, EE, NDF, and ADF, which were reduced as the inclusion of *Sargassum* increased from 10.48 to 9.36%; from 92.54 to 82.58%; from 1.34 to 1.11%; from 76.70 to 60.63%; and from 41.61 to 34.28%, respectively (Table 1 and Table 2).

**Table 1:** Chemical composition of *Sargassum* and Stargrass.

Ingredients	DM (%)	Ash (%)	OM (%)	CP (%)	NDF (%)	ADF (%)	EE (%)	Lignin (%)	NFC (%)	TFC	TT
Stargrass	93.46	7.47	92.53	10.48	76.70	41.61	1.34	ND	4.01	ND	ND
Sargassum	87.76	40.07	59.93	6.73	23.12	17.18	0.57	20.35	29.51	0.07	0.04

NFC: non-fibrous carbohydrates = 100 − (CP + NDF + EE + Ash); TFC: Total phenolic compounds; TT: tannins totals; ND: not determined.

**Table 2:** Ingredients and chemical composition of dietary treatments.

Components	Treatments			
	TC	T10	T20	T30
Inclusion (%)				
Stargrass	100	90	80	70
*Sargassum*	0	10	20	30
Chemical composition (%)				
Dry matter	93.46	92.87	92.56	91.67
Organic matter	92.54	88.71	87.44	82.58
Ash	7.46	11.29	12.56	17.42
Cru de protein	10.48	10.10	9.73	9.36
Ether extract	1.34	1.26	1.19	1.11
Neutral detergent fiber	76.70	71.34	65.98	60.63
Acid detergent fiber	41.61	39.17	36.72	34.28

Differences were observed in the concentrations of macro and micro minerals. Cl, S, Ca, K, and Zn (103, 5.97, 88.73, and 285.70 g/kg and 15,900 mg/kg) were high in *Sargassum*, reaching twice or even nine times higher than the contents in Stargrass (11.37, 1.60, 43.53, and 87.73 g/kg and 866.67 mg/kg). The elements Al and As (500 and 530 mg/kg) were only found in *Sargassum* (Table 3).

**Table 3:** Macro and micro mineral contents of Stargrass and pelagic *Sargassum*.

	Ingredient	
	Stargrass	*Sargassum*
Macrominerals (g/kg)		
Cl	11.37	103.20
Na	6.90	1.80
Mg	0.90	0.0
S	1.60	5.97
Ca	43.53	88.73
P	0.60	0.0
K	87.73	285.70
Microminerals (mg/kg)		
Fe	10,033.33	1600
Mn	233.33	100.00
Zn	866.67	15,900
Cu	66.67	0.0
Al	0.0	500
Si	3333.33	1266.67
Heavy metals (mg/kg)		
As	-	530
F	ND	ND
Cd	ND	ND
Cr	ND	ND
Pb	ND	ND
Hg	ND	ND

All values represent the mean of triplicates. NA: not determined.

### 3.2. Total Gas Production and Characteristics of In Vitro Fermentation

The pH was similar between treatments at 24 h of incubation (*p* = 0.0875). At 48 h of incubation, there was a dose-dependent effect, where the lowest pH value (*p* < 0.0001) was recorded at T30; while at 72 h, the lowest value (*p* = 0.0507) was observed in T20 compared to the other levels containing *Sargassum*, but they were similar to the control values (TC). Despite the differences in the pH values reported in the present study, they are still within the optimal range (5.66–7.47) (Table 4).

**Figure 1:**
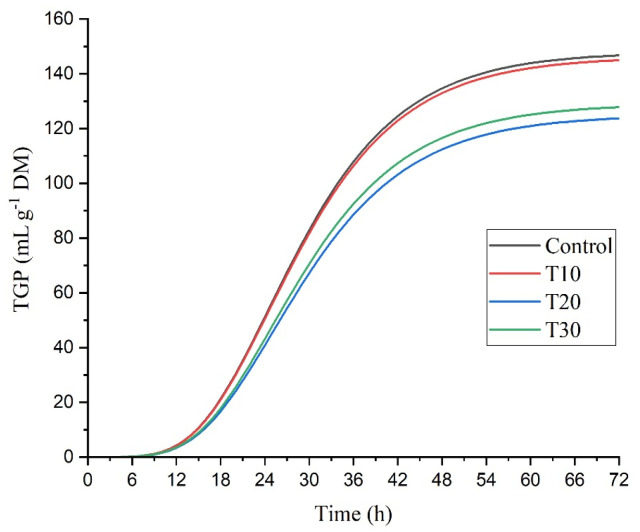
Effect of *Sargassum* inclusion level on in vitro total gas production (TGP) during 72 h of incubation.

The total gas production (TGP) had differences at 48 h (*p* = 0.0137), and the lowest production was found at T20 with respect to TC (127.56 vs. 107.77 mL/g DM) (Table 5 and Figure 1). The DM degradability (DMD) was affected by the addition of *Sargassum* at 24, 48, and 72 h of incubation (*p* = 0.0105; *p* = 0.0056; *p* = 0.0055). At 24, the treatments T10 and T30% registered the highest DMD (*p* = 0.0105) with respect to the control (32.22 and 33.90 vs. 24.67%). At 48 h of incubation, T30 was the one that showed the highest (*p* = 0.0056) degradability (37.91 vs. 29.36%), and the other treatments were similar to each other and to the control. At 72 h of incubation, T20 and T30 had a higher DMD (*p* = 0.0055), while T10 was similar to the control. Regarding the OM degradability (OMD), differences were observed at 24 and 48 h of incubation (*p* = 0.0487, *p* = 0.0141); the highest values were recorded at T20. At 72 h; and no differences were observed (*p* = 0.2077) in OMD.

### 3.3. Protozoa Population

Total protozoa population was similar between treatments at 24 h of incubation (*p* = 0.1215), while at 48 and 72 h, there was an observed effect of the treatments (*p* = 0.0291; *p* = 0.0130), namely, T20 at 48 h and T30 at 72 h, i.e., those that showed the lowest concentrations of protozoa (Table 4).

## 4. Discussion

### 4.1. Total Gas Production and In Vitro Fermentation Kinetics

The inclusion of algae in ruminant diets modifies digestion, fermentation kinetics, proteolysis, and nitrogen metabolism, which causes changes in rumen microbial communities [8,16,22,33,34,35]. In this regard, ruminal pH is considered the main factor that influences microbiome and the degradability of DM and OM, the concentration of NH_3_, and molar proportions of volatile fatty acids [9,20]. In this study, although the pH was affected by *Sargassum* inclusion (*p* < 0.0001; *p* = 0.0507), the values obtained were in the optimal range for adequate microbial growth (5.5–7.5) (Table 5). Therefore, the inclusion of *Sargassum* in the basal substrate could provide a stable and adequate environment for rumen microorganisms’ growth [23,35,36]. Compared with control, at 48 h of incubation, the highest pH (*p* < 0.0001) was obtained with T10; however, all other values were above a pH of 6, results that do not coincide with those reported in other studies evaluating *Sargassum fusiform* and *Sargassum fulvellum* at 10% inclusion to a substrate based on Timothy grass (*Phleum pretense*), where no differences in pH were observed [20,35]. On the other hand, Choi et al. [23] evaluated extracts of five species of algae added at a level of 5% and found that the highest pH at 72 h was obtained with *Sargassum fusiform*, and pH values recorded at 24, 48, and 72 h were above 6, which coincides with those obtained in the present study.

Total gas production is related to substrate degradability, VFA production, and microbial growth in the rumen [37,38,39]. Therefore, the addition of algae in the substrate is directly related to an increase in the populations of bacterial species such as *Fibrobacter succinogenes*, and *Ruminococcus flavefaciens*, which are responsible for degrading dietary fiber [23,24]. In this study, a reduction in TGP was observed with T20 at 48 h incubation (*p* = 0.0137). However, it did not influence DMD, and this was affected by the level of *Sargassum* in the basal substrate, with T30 being the one that showed the highest DMD at all incubation times (*p* = 0.0105; *p* = 0.0056; *p* = 0.0055), showing that there was a linear trend for DMD with the increase in the inclusion of *Sargassum*. These results coincide with those of Widiawati and Hikmawan [40], who observed a linear increase in DMD with increasing inclusion at 48 h of incubation of *Eucheuma cottonii.*

The results of the present study also agree with those obtained by Choi et al. [15], who observed a dose-dependent effect on DMD with the inclusion of increasing levels of *U. pinnatifida* to a Timothy grass-based substrate. For their part, Choi et al. [24] argued that the greater degradability of the DM obtained in treatments with brown algae was due to the increase in the abundance of fibrolytic bacterial populations. In this study, T10 at 48 and 72 h of incubation did not show differences compared with the control, and these results are similar to those reported by Choi et al. [20] and Choi et al. [35] with *Sargassum fusiform* and *Sargassum fulvellum* at 10% inclusion, where they found no differences in DMD compared to the control. Maia et al. [16] evaluated three species of algae (*Ulva rigida, Gracilaria vermiculophylla*, and *Saccharina latissima*) as supplements added at 25% to a basal substrate and reported increases in DMD with all algae compared to the control. However, the findings of Rjiba-Ktita et al. [41], with increasing inclusion levels of green algae up to 40% to concentrated feed as substrate, yielded a linear decrease in DM degradability with increasing inclusion levels of *Ulva lactuca* and *Chaetomorpha linum*.

The OMD was influenced by the addition of *Sargassum* to the basal substrate; however, unlike the DMD, the highest value was observed at T20 (*p* = 0.0141). Some studies evaluating the composition and chemical characterization of pelagic *Sargassum* reported ash and OM contents of 46.94 and 30.61%, respectively [24,39]. In this study, the ash concentration and consequently low OM level of *Sargassum* was expected to negatively affect the OMD, which contains 40.07 and 59.93% ash and OM, respectively (Table 1). However, this situation did not occur; therefore, it is possible that high levels (20–30% DM) of inclusion of this unconventional ingredient can be used under in vitro conditions. The results observed in this study agree with those reported by Maia et al. [16], who included 25% *Saccharina latissima* to a mixed total ration where OMD was increased.

For their part, Widiawati and Hikmawan [40] observed a linear increase in OMD with increasing inclusion of *Eucheuma cottonii* at 48 h of incubation added to a substrate based on elephant grass (*Pennisetum purpureum*). The improvement in the digestibility of nutrients is due to the bioactive compounds (i.e., polysaccharides such as fucoidan, alginate, laminarin and mannitol) from brown algae, which can favor changes in metabolic pathways with an increase in fibrolytic activity [20,23]. These polysaccharides cause changes in ruminal microbiome because they are highly available for microbial growth and favor the production of acetate and butyrate, which are directly related to fiber degradation in the rumen [15,35]. In this sense, the degradability of nutrients is influenced by algal species [37]. This species effect is directly related to the chemical composition and secondary metabolite content of the algae, which includes polysaccharides, polyphenolic compounds, halogenated compounds, minerals, and fatty acids, which confer various biological properties [14,16,33,41,42,43,44,45].

The composition of the cell wall influences in vitro degradability, apparent digestibility, and availability of nutrients from algae as feed for ruminants [46]; however, there is also an interaction effect between algae species and harvesting season on in vitro nutrient degradability [38]. Regarding pelagic *Sargassum*, according to the study by Saldarriaga-Hernandez et al. [47], the harvest season influences the composition of carbohydrates, proteins, and total phenolic compounds due to several factors, such as light intensity and solar radiation, that affect the growth of these algae.

This study was carried out with the purpose of using non-conventional additives or ingredients in ruminant feed and verifying their effects on rumen fermentation kinetics. The results of this research contribute valuable information to reduce dependence on grains for formulating diets, which would reduce production costs [1,5,8,48,49,50,51,52]. However, it is convenient to carefully analyze the level of inclusion of these ingredients according to the species of seaweed, since the purpose is not to negatively affect fermentation parameters that could affect productivity and animal performance. This is related to changes in metabolic hydrogen (H) fluxes in ruminal fermentation and in the post-absorption metabolism of the animal, caused by CH_4_ inhibitors [53,54,55]. In ruminants, reductions in the intake and digestibility of diets with seaweed have been reported; this is due to the increase in the mineral contents of the diet and the increase in H levels in the rumen due to the inhibition of methanogenesis [56,57,58].

### 4.2. Population of Protozoa

Marine macroalgae contain a wide variety of bioactive compounds depending on the species; among these, we can highlight bromoform and phlorotannin that have antimicrobial properties [1,21,59,60]. According to Choi et al. [23], phlorotannin modify the abundances of cellulolytic bacteria, methanogenic archaea, and methanogens associated with ciliate protozoa. The antibacterial mechanism of action of these phenolic compounds is mediated by their ability to affect cell wall permeability. Phlorotannin change the shape of the bacterial cell membrane, leading to cell lysis; nevertheless; they also suppress bacterial reproduction through their union with bacterial proteins, RNA and DNA, inhibiting cell replication [61]. In this study, effects were observed at 48 and 72 h of incubation on the population of protozoa, which decreased in treatments T20 and T30 (Table 5). Although the reduction of protozoa was not linear, the inhibitory effect of brown algae on the protozoa population of ruminal fluid was demonstrated. These results agree with those obtained by Choi et al. [20], who evaluated *Sargassum fusiform* at levels of 1 to 10%, and with those reported by Prayitno and Hidayat [62], who evaluated *Sargassum* sp. at levels of 1 to 5%, which were accompanied by a CH_4_ reduction of more than 40 and 80%, respectively. They also agree with the study by Belanche et al. [14] when evaluating *Ascophyllum nodosum* and *Laminaria digitata* added to 5% DM, which showed reductions in the concentration of methanogens and protozoa without affecting the bacterial population and anaerobic fungi.

Other studies with red algae have reported similar results, such as the study by Widiawati and Hikmawan [40], which evaluated *Eucheuma cottonii* with inclusion levels of 4, 8, and 12%. They observed a linear decrease in the population of protozoa and in the concentration of CH_4_ in the ruminal liquid with the increase in the dose. Roque et al. [8] reported reductions in the abundance of methanogens in ruminal fluid and, consequently, a 95% reduction in CH_4_ production with the addition of 5% *Asparagopsis taxiformis* to a good quality substrate. Contrary to these studies, Molina-Alcaide et al. [37], when evaluating various species of red algae (*Mastocarpus stellatus*, *Palmaria palmata*, and *Porphyra* sp.), reported no effects on the microbial population.

Some studies with brown seaweed extracts have reported differential effects, for example, Choi et al. [24] reported an increase in the population of ciliate protozoa with *Sargassum fusiform*, while with *Undaria pinnatifida* and *Sargassum fulvellum*, there was a decrease. This effect was also reported by Belanche et al. [18], who demonstrated antiprotozoal activity with *Ascophyllum nodosum* compared to *Laminaria digitata* which did not show antiprotozoal effect. These differential effects are probably due to differences in the number of polyphenolic compounds, especially in the concentration of phlorotannin present in each species [14]. Based on the above, and on the results obtained in this study, it is important to pay special attention to the concentration of polyphenols, the chemical structure, and the molecular weight of phlorotannin in the different species of brown algae to know their mode of action [61]. In addition to the above, the characterization of the sulfated polysaccharides from pelagic *Sargassum*, based on their bioactive properties in ruminants, is another point of interest since there are no studies in this regard so far.

For the reasons stated, it is necessary to carry out more studies to determine the composition of fatty acids, minerals, potentially toxic compounds, and secondary metabolites in pelagic *Sargassum*, and their effects on the microbial communities of the rumen. Furthermore, further in vitro work with different base substrates and inclusion levels is of vital importance [63,64]. Moreover, long-term in vivo studies are required with different species of ruminants, and with different feeding regimens to verify and rule out harmful effects of *Sargassum* on the health of animals and on the quality of meat and milk [49,56,57,58,65,66,67,68,69]. The findings and their implications should be discussed in the broadest possible context. Future research directions may also be highlighted.

## 5. Conclusions

Pelagic *Sargassum* has great potential as ruminant feed. The results of this in vitro study showed that the inclusion of up to 30% pelagic *Sargassum* in hay-based substrates from tropical grasses does not have negative effects on rumen fermentation kinetics, nor on the degradability of dry matter and organic matter. The use of this unconventional natural resource in ruminant production systems would have important economic benefits since it provides a route for the management of marine algae residues in the Mexican Caribbean. This would reduce the negative impact of pelagic *Sargassum* on the tourism sector, which is the primary source of income for families living in is region.

## Figures and Tables

**Table 4:** Effect of *Sargassum* inclusion level on pH and total concentration of protozoa in ruminal fluid.

Time (h)	Treatments				SEM	*p* Value
	TC	T10	T20	T30		
pH						
24	6.55	6.70	6.62	6.62	0.037	0.0875
48	6.42 ^bc^	6.60 ^a^	6.47 ^b^	6.37 ^c^	0.021	<0.0001
72	6.37 ^ab^	6.32 ^ab^	6.30 ^b^	6.42 ^a^	0.029	0.0507
Total protozoa (×10^5^ cel/mL) log_10_						
24	6.23	6.85	6.47	6.43	0.125	0.0986
48	6.80 ^a^	6.49 ^ab^	6.23 ^b^	6.49 ^ab^	0.075	0.0262
72	6.37 ^a^	6.03 ^ab^	6.34 ^a^	5.65 ^b^	0.086	0.0120
Total Entodinium (×10^5^ cel/mL) log_10_						
24	3.41	4.02	3.65	3.45	0.144	0.1157
48	3.98 ^a^	3.66 ^ab^	3.41 ^b^	3.51 ^b^	0.079	0.0258
72	3.55 ^a^	3.21 ^ab^	3.36 ^a^	2.82 ^b^	0.082	0.0131
Holotrichs (×10^5^ cel/mL) log_10_						
24	2.82	2.82	2.82	2.97	0.075	0.4789
48	2.82	2.82	2.82	2.97	0.075	0.4789
72	2.82	2.82	2.97	2.82	0.075	0.4789

^a,b,c^ Columns with literals different indicate difference statistic (*p* < 0.05). SEM: Standard error of the mean; n = 4. Treatments: TC = control (Stargrass); T10 (Stargrass with 10% *Sargassum*); T20 (Stargrass with 20% *Sargassum*); T30 (Stargrass with 30% *Sargassum*).

**Table 5:** Effect of *Sargassum* inclusion level on in vitro total gas production, dry matter degradability, and organic matter degradability.

Time (h)	Treatments				SEM	*p* Value
	TC	T10	T20	T30		
Total gas production (mL/g DM)						
24	51.88	55.79	52.98	55.37	2.867	0.7350
48	127.56 ^a^	115.58 ^ab^	107.77 ^b^	113.39 ^ab^	3.578	0.0137
72	148.29	146.21	125.27	129.85	6.955	0.0883
Dry matter degradation (%)						
24	24.67 ^b^	32.22 ^a^	31.47 ^ab^	33.90 ^a^	1.675	0.0105
48	29.36 ^b^	31.22 ^b^	35.25 ^ab^	37.91 ^a^	1.461	0.0056
72	30.07 ^b^	35.14 ^ab^	36.60 ^a^	37.56 ^a^	1.256	0.0055
Organic matter degradation (%)						
24	21.29 ^b^	23.13 ^ab^	25.45 ^a^	22.76 ^ab^	0.818	0.0487
48	26.60 ^ab^	23.28 ^b^	30.26 ^a^	28.26 ^ab^	1.139	0.0141
72	26.76 ^a^	31.44 ^a^	30.39 ^a^	29.36 ^a^	1.346	0.2077

^a,b^ Columns with different literals indicate statistical difference (*p* < 0.05). SEM: Standard error of the mean; *n* = 4. Treatments: TC = control (Star grass); T10 (Star grass with 10% *Sargassum*); T20 (Star grass with 20% *Sargassum*); T30 (Star grass with 30% *Sargassum*).

## Data Availability

Data presented in this study are available on request from the corresponding author.
